# Abdominal Muscle Fasciculations and Amyotrophic Lateral Sclerosis Diagnosis in a Patient Unable to Perform Sit-Ups

**DOI:** 10.7759/cureus.23498

**Published:** 2022-03-25

**Authors:** Hiroshi Hori, Takahiko Fukuchi, Hitoshi Sugawara

**Affiliations:** 1 Internal Medicine, Minamiuonuma City Hospital, Niigata, JPN; 2 Department of Comprehensive Medicine, Division of General Medicine, Saitama Medical Center, Jichi Medical University, Saitama, JPN

**Keywords:** sit-ups, motor neuron disease, muscle ultrasonography, fasciculations, amyotrophic lateral sclerosis

## Abstract

While performing sit-ups, a 70-year-old man was unable to lift his upper body. The abdominal skin reflex was absent, and abdominal ultrasonography showed intermittent, irregular, and localized muscle twitches of the abdominal muscles. Further, electromyography (EMG) detected widespread fasciculations. Amyotrophic lateral sclerosis (ALS) was diagnosed. Muscle ultrasonography (MU) is useful in detecting fasciculations. This technique allows for repeated non-invasive imaging and the assessment of an expansive range of muscles in real-time. It also detects deep abdominal muscles, which are difficult to assess using EMG. MU is particularly beneficial to patients with atypical ALS who experience truncal muscle weakness.

## Introduction

Amyotrophic lateral sclerosis (ALS) is associated with a poor prognosis [1,2]. The five-year survival rate is 29%, and the median survival from symptom onset is approximately 3.5 years [3]. At present, no drug has effectively and reliably slowed the progression of ALS [1]. Early diagnosis, comprehensive rehabilitation, and nutritional interventions help maintain the quality of life in ALS patients. Moreover, early diagnosis may allow timely participation in clinical trials and prevent unnecessary diagnostic workup. Thus, early diagnosis of ALS is very important.

ALS associated with trunk muscle weakness is rare (6.1%) [4], which may cause a delayed diagnosis. There are no specific markers for ALS diagnosis, and it is diagnosed by the characteristics of clinical symptoms, examination findings, electrophysiological examination, and exclusion of other diseases. The presence of fasciculations also contributes to the ALS diagnosis [5-7].

Electromyography (EMG) is the standard method for detecting fasciculation. However, EMG is difficult to use for abdominal and thoracic muscles as it can be invasive, it is difficult to simultaneously examine a wide range of muscles, and it is not suitable for short-term repetition. In contrast, muscle ultrasonography (MU) has been reported to be an excellent alternative for detecting fasciculation [8].

Herein, we report a case of ALS, diagnosed by the detection of fasciculation by ultrasonography, with the chief complaint of abdominal muscle weakness. We then describe the usefulness of MU as an auxiliary test in ALS.

## Case presentation

A 70-year-old man presented to the hospital with difficulty in returning to an upright position after bending down. He had experienced this symptom for six months. The patient would become easily fatigued while playing tennis, which was his hobby, and thus, started going to the gym to increase his strength. However, he was unable to lift his upper body while performing sit-ups.

**Figure 1:**
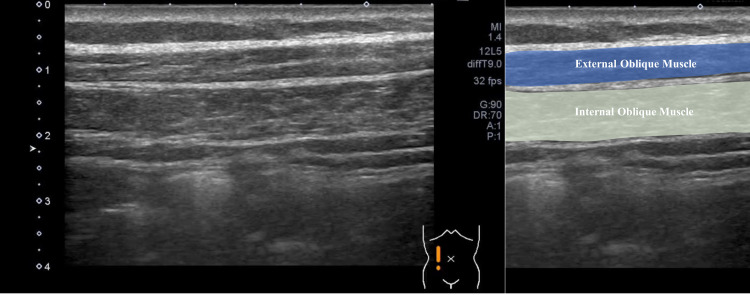
Muscle ultrasonography. Long-axis image of the oblique muscles.

**Figure 2:**
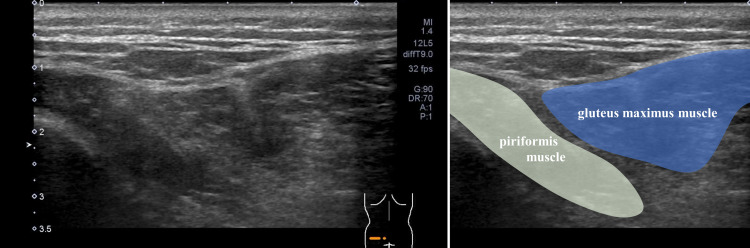
Muscle ultrasonography. Short-axis image of the gluteal and piriformis muscles.

Manual muscle testing of the extremities performed at the initial examination revealed that the patient’s muscle strength was maintained. Examination revealed no abnormalities in the patient's cranial nerves. He had positive signs for the Trömner reflex in the left upper extremity and the Wartenberg thumb adduction reflex bilaterally; the tendon reflex of the extremities was exaggerated. However, the patient was unable to lift his upper body while performing sit-ups. Abdominal skin reflexes on both sides disappeared upon examination, and ultrasonography showed temporal, irregular, and localized muscle twitches of the abdominal oblique muscle (Video 1, Figure 1) and the gluteus muscle (Video 2, Figure 2).

**Video 1:** Long-axis image of the oblique muscles. Long-axis image of the oblique muscles using linear ultrasonography (frequency: 10 MHz). Intermittent, irregular, and localized muscle twitches of the abdominal oblique muscles were observed.

**Video 2:** Short-axis image of the gluteal and piriformis muscles. Short-axis image of the gluteal and piriformis muscles using linear ultrasonography (frequency: 10 MHz). Localized muscle twitches of the gluteal and piriformis muscles were observed.

We considered spinal muscular atrophy, ALS, peripheral neuropathy, spinal cord diseases such as syringomyelia, and muscle diseases as differential diagnoses.

Magnetic resonance imaging of the head, cervical, and thoracic cords, as well as nerve conduction velocity, were normal. In addition, EMG detected widespread fasciculations and denervation in the trapezius, biceps brachii, and quadriceps. From these results, spinal cord disease, peripheral nerve disease, and muscle disease were ruled out, and a high possibility of motor neuron disease was considered. The survival of motor neuron 1, telomeric (SMN1) gene contained no mutations, which are characteristic of spinal muscular atrophy. No abnormalities were found in the respiratory function test (spirometry).

Subsequently, the patient developed muscle weakness in the lower limbs, requiring him to use a walking stick. There was no evidence of frontotemporal dementia. Ten months after the onset of symptoms, he met the Awaji criteria [9] and was diagnosed with sporadic ALS by a neurologist.

## Discussion

Fasciculation is a small, localized, involuntary muscle contraction that lasts for 4.5-69.3 ms and exhibits signs of lower motor neurons [10]. In particular, the presence of fasciculations can be detected by EMG and is very useful for diagnosing motor neuron diseases, such as ALS. Aside from EMG, MU is useful for the detection of fasciculations [8].

Advantages of MU compared with EMG in diagnostic surveillance for fasciculations include its non-invasive nature, the ability to sample from a larger area of the muscle in real-time, and ease of monitoring over time. During an examination, it is necessary to rest the examined muscle to distinguish it from contraction fasciculation. In this respect, non-invasive ultrasonography has an advantage over needle EMG. In particular, MU becomes useful when detecting deep and abdominal muscles, which are difficult to assess with EMG. In addition, MU is more sensitive than EMG for detecting fasciculations in patients with ALS [11]. Moreover, combining these two techniques is more sensitive than one procedure alone [12]. MU has excellent sensitivity to detect fasciculation and is a useful technique for early suspicion of motor neuron disease when combined with EMG. Furthermore, there are many reports that MU is useful for diagnosing ALS. For example, a fasciculation ultrasonography score, indicating the number of muscles with fasciculations assessed by MU, has been effective in ALS diagnosis. Specifically, sensitivity, specificity, and the area under the curve are 92%, 100%, and 0.97, respectively [13]. In addition, MU is useful for differentiating between ALS (a positive likelihood ratio of 6.17) and non-ALS patients (a negative likelihood ratio of 0.04) [14]. The fasciculation grade, assessed by MU, was higher in ALS patients than in non-ALS patients, and the distribution pattern was more easily detected in the proximal limbs of ALS patients [15]. Several MU findings, such as the extent and frequency of fasciculations, the decrease in muscle thickness, and the increase in echogenicity, are useful for estimating ALS progression and prognosis [16].

MU has some limitations. MU can only detect fasciculation and is not a definitive test for ALS diagnosis. ALS is a disease that can only be comprehensively diagnosed from the clinical course, medical examination findings, various tests, and exclusion of other diseases, and the detection of fasciculation is only one of the diagnostic factors. Attempts to use MU for ALS diagnosis are relatively new, and specific ultrasonic procedures and measurement methods have not been standardized [17]. Furthermore, there are few high-quality studies differentiating between benign fasciculation and other diseases [17].

At present, EMG is the key diagnostic test for ALS, and MU remains an adjunct diagnostic method [18,19]. For the definitive diagnosis of motor neuron disorders, it is necessary to confirm that there is a neurogenic change in the background by performing an EMG.

However, in the future, if standardization in MU procedures progresses and the validity of diagnostic criteria using MU in ALS diagnosis is positively evaluated, MU may be incorporated into diagnostic criteria as an alternative to EMG as a modality to detect fasciculation. MU is useful for detecting fasciculation in patients in whom it is difficult to perform EMG, such as patients with atypical ALS, who present with weakness of the trunk muscles, such as the abdominal muscles, as in this case. In such cases, MU findings are the basis for positively suspecting motor neuron disease.

## Conclusions

If a patient is unable to perform sit-ups, a motor neuron disease, which develops as trunk muscle weakness, should be considered as one of the differential diagnoses. Further, fasciculations are important for diagnosing motor neuron diseases presenting as a lower motor neuron sign. Finally, detection of fasciculations by ultrasonography waves is non-invasive, can be easily and repeatedly performed, and is particularly useful for probing trunk muscles, which are difficult to examine using EMG.
